# A semi-automated pipeline for morphological analysis of myonuclei along single muscle fibers

**DOI:** 10.1016/j.isci.2026.116961

**Published:** 2026-07-29

**Authors:** Esben T. Schroeder, Helia G. Megowan, Madeline Luu, Adam Shuaib, Adam C. Fries, Jake Searcy, Hans C. Dreyer

**Affiliations:** 1 Department of Human Physiology, University of Oregon, Eugene, OR 97403, USA; 2 Department of Computer Science, University of Oregon, Eugene, OR 97403, USA; 3 Department of Data Science, University of Oregon, Eugene, OR 97403, USA; 4 Department of Neuroscience, University of Oregon, Eugene, OR 97403, USA; 5 Genomics & Cell Characterization Core Facility, University of Oregon, Eugene, OR 97403, USA

**Keywords:** ImageJ, muscle, pipeline, automation

## Abstract

Manual quantitation of skeletal muscle myonuclear number, spatial orientation, and morphology is time-consuming and subject to error and bias. To overcome these limitations, we developed and validated a semi-automated, quantitative, and reproducible image-analysis pipeline. The workflow combines FIJI-based preprocessing with custom Python scripts to process immunohistological images of individual muscle fibers, enabling high resolution and scalable quantification of nuclei. The analyses incorporate morphometric parameters including nuclear position, shape, and three-dimensional orientation, as well as centroid-to-skeleton distance and nearest-neighbor relationships to capture spatial patterns of myonuclear organization along the fiber. Outputs include per-fiber and biopsy-level summaries integrated with IMARIS metrics. This semi-automated approach provides a robust and efficient platform for high-throughput analysis of myonuclear number and structural features across large single fiber datasets.

## Highlights

• A new method automates myonuclear morphological analysis at scale
• Quantitative assessment of nuclear count, shape metrics, and spatial arrangement
• Provides a high-throughput and customizable analysis platform of single muscle fibers

## Graphical abstract

**Figure:**
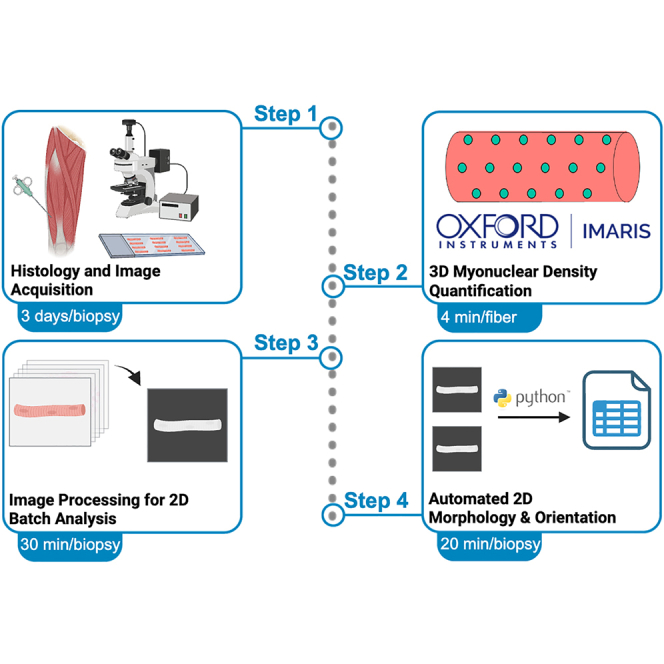

## Teaser

Cell biology; Computational bioinformatics

## Introduction

Histological analysis of skeletal muscle tissue is helpful in understanding the relationship between anatomical structure and physiological function.^1^ Immunohistochemical techniques, which employ labeled antibodies to localize specific proteins, are often integrated with advanced imaging methods to provide detailed insight into the structural organization of muscle tissue. Muscle fibers exhibit remarkable plasticity in response to internal and external stimuli, leading to morphological adaptations such as increases in muscle fiber cross-sectional area (CSA), changes in mitochondrial and myonuclear content, and fiber type transitions. These structural adaptations, visualized histologically, are commonly used as proxies for functional capacity.

A prevailing method in the muscle field is to quantify structural and positional changes histologically using tissue sections cut perpendicular to the long axis of the fibers. This cross-sectional approach allows more individual cells/fibers (each fiber is a single muscle cell) to be analyzed, providing greater coverage of the muscle being studied and therefore better representation. However, if information about myonuclei number and structure is of interest, the cross-sectional approach may under sample given the multinucleated syncytial nature of muscle cells. For example, along a one millimeter length segment of muscle cell there are 100 10 µm-thick cross sections, and if the muscle sample obtained from biopsy are 3 cm to 5 cm in length,^2^^,^^3^ this constitutes 3000 (i.e., 3 cm) to 5000 (i.e., 5 cm) 10 µm-thick sections. In other way, one 10-μm-thick cross section represents 0.00033% (1/3,000) to 0.0002% (1/5,000) of the approximate 3–5 cm length of single muscle fiber cells^2^^,^^3^—and averages out to about 2–3 myonuclei per 7- to 10-μm-thick cross section. Conversely, hundreds to thousands of myonuclei can be included when sampling lengthwise along the long axis of single muscle fibers, improving statistical power, confidence, and rigor for studies that center on the changes to myonuclear structure and function.

Precise quantification of myonuclei is critical for advancing our understanding of skeletal muscle biology, particularly in relation to adaptations such as myonuclear accretion. This is especially relevant to the ongoing assessment of the myonuclear domain hypothesis, which posits that each myonucleus governs a defined cytoplasmic volume.^4^^,^^5^ Traditionally, myonuclear domain has been assessed using cross-sectional tissue samples; however, recent studies suggest that myonuclear accretion correlates more strongly with myofiber surface area rather than fiber volume alone.^6^^,^^7^^,^^8^ Beyond myonuclear accretion, additional parameters that may be of interest to the muscle biology field—including myonuclear shape, spatial orientation along the fiber’s long axis, and the distribution and/or clustering of myonuclei of similar shape—are emerging as important indicators of muscle cell regulation and remodeling.^9^^,^^10^^,^^11^^,^^12^

Motivated by the abovementioned discussion, in this study, we developed a semi-automated image analysis pipeline for immunohistological images of isolated single muscle fibers that allows:

1. quantification of myonuclear number along the length of individual fibers,
2. classification of each myonucleus into predefined morphological categories,
3. assessment of local clustering by quantifying the number of similarly shaped myonuclei within a fixed radius, and
4. quantification of the long-axis orientation of each myonucleus relative to the long axis of the single fiber.

To our knowledge, this is the first study to integrate these analytical features into a single pipeline using widefield microscopy—a platform broadly accessible in core facilities and individual laboratories. This approach allows high-throughput analysis of large composite images, yielding rich datasets on nuclear organization and spatial relationships along individual myofibers. The method has broad applicability in biomedical research and may help uncover myonuclear structure-function relationships within various physiological and pathological contexts across the lifespan.

## Results

### Ethical approval

The study was approved by the University of Oregon Institutional Review Board (STUDY00000938) and conducted in accordance with the Declaration of Helsinki ethical principles. All subjects gave informed written consent prior to study participation.

### Demographics

Biopsy tissue from 9 different human subjects was used for various validations of methods in this paper. Demographic information is presented in Table 1.

**Table 1:** Subject characteristics

	Male (*n* = 5)	Female (*n* = 4)
Age (years)	22 ± 1.4	27 ± 5.0
Height (m)	1.79 ± 0.04	1.65 ± 0.05
Weight (kg)	75 ± 4.03	72 ± 16.6
BMI (kg/m^2^)	23 ± 1.97	26 ± 4.88

Data are presented as mean ± SD.

### Biopsies

Skeletal muscle biopsies were performed on human vastus lateralis in a distal to proximal sequence (if multiple biopsies) and in the fasted state. For serial biopsies, the biopsy needle was oriented laterally from and perpendicular to the long axis of the femur, with the biopsy needle window/opening situated in the mid-substance of the muscle tissue. Serial biopsies were thus obtained from the mid-substance of the vastus lateralis muscle that are 3 cm or more apart (and not angled).^13^^,^^14^ Biopsy samples were obtained using a Bergström needle with a 5 mm internal diameter with manual suction. Each biopsy yielded approximately 300 mg of tissue (range ∼100–500 mg) with fiber bundles approximately 2–7 mm in length. Our goal for single fiber analysis is to obtain a minimum of 20 individual muscle cells for analysis per biopsy.

### Single fiber isolation and immunohistochemistry

Immediately following biopsy collection, tissue sections with the longest fiber bundles were teased apart and placed in a 0.6 mL Eppendorf tube containing 500 μL 4% paraformaldehyde (PFA) for 48 h at 4°C. Fixed fiber bundles were then placed in a 0.6 mL Eppendorf tube containing PBS and kept in the dark at 4°C until further processing.

To begin tissue processing, the biopsy sample was placed in a 3-cm-diameter glass dish containing a small amount of PBS to prevent drying. Under a benchtop stereomicroscope, the fixed sample was teased apart with forceps into smaller bundles (final size being ≤30 fibers), with care being taken to avoid damaging or tearing the fibers. Once separated, the next step is to remove all extracellular components from the sample to further isolate the single fibers. To achieve this, the tissue bundles were transferred into a 1.5 mL Eppendorf tube containing 750 μL of 40% NaOH, then placed on a tube rotator at moderate speed for 2 h to facilitate digestion of the connective tissue between the fibers. Following digestion, NaOH was removed by filtering the bundle through a 40 μm mesh strainer and repeatedly washed with 1× PBS (10–12 total). Fibers were then collected from the strainer using forceps and transferred into a 0.6 mL Eppendorf tube containing 300 μL 1× PBS until further processing.

Following isolation, fibers were permeabilized in a 1.5 mL Eppendorf tube containing 750 μL 1% Triton X-100 for 10 min on the rotor wheel. Permeabilized fibers were then centrifuged at 13,000g for 3 min to pellet fibers. After centrifugation, supernatant was removed, and fibers were incubated in primary antibody mouse anti-slow myosin (cat. no. M8421, Sigma-Aldrich, Darmstadt, Germany), 1:200 in PBS for 90 min at room temperature. Following incubation in the primary antibody, fibers were washed three times for 3 min in 1% Triton X-100, centrifuged (13,000 × g for 3 min to pellet fibers). Fibers were then transferred to a 1.5 mL Eppendorf tube containing the secondary antibody Alexa Fluor 555 goat anti-mouse IgG1 (cat. no. A-21127, Invitrogen, Carlsbad, CA, USA), 2:500 in 250 μL PBS and 250 μL 1% Triton X-100 for 1 h at room temperature. Following incubation in the secondary antibody, fibers were again washed three times for 3 min in 1% Triton X-100, with centrifugation. Fibers were then placed in 100 μL of PBS with 1 μL of 100 μM DAPI (DAPI hydrochloride, cat. no. D1306, Invitrogen, Carlsbad, CA, USA) for 5 min.

Following staining, fibers were transferred to glass microscope slides and carefully separated with forceps to avoid overlap. Vectashield Mounting Medium (H-1000, Vector Laboratories, Newark, CA, USA) was applied to maintain separation and prevent fading. Slides were then mounted with a glass coverslip and sealed with clear nail polish.

### Image acquisition

All slides of fluorescently labeled, fixed single fibers were imaged using a Leica DMi8 Thunder wide field fluorescence microscope equipped with Leica’s LED8 illuminator and K8 camera (6.5 μm pixels). Fibers were first visually characterized as either type I (myosin heavy chain [MyHC]-1^+^) or type II (MyHC-1^−^) using the HC PL FLUOTAR 10×/.32 objective in the TRITC channel (excitation and emission filters: 554/24 and 594/32) at 25% illumination intensity and an exposure time of 200 ms (Figure 1).

**Figure 1:**
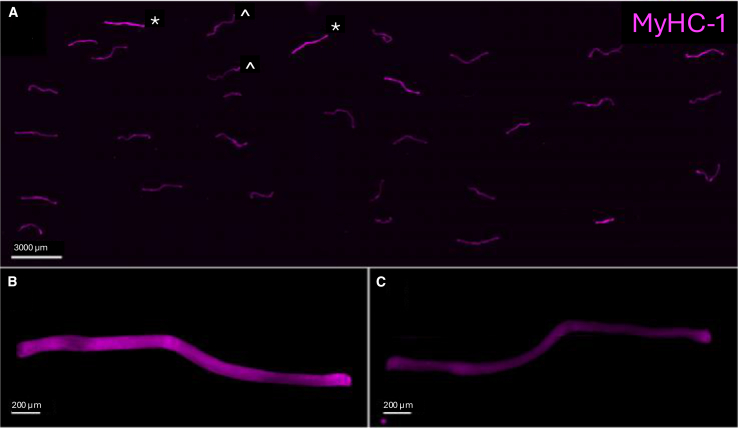
Fiber type characterization (A) Representative merged Tile Scan image of fiber type characterization for a slide of fixed muscle fibers stained with myosin heavy chain I (MyHC-1); fibers are characterized based on fluorescence intensity under the TRITC channel (555 nm). Type I fibers (∗) fluoresce at a greater intensity than type II fibers (ˆ). Scale bars, 3,000 μm. (B) Representative type I muscle fiber. Scale bars, 200 μm. (C) Representative type II muscle fiber. Scale bars, 200 μm.

Once identified based on fiber type, image stacks of myonuclei on entire single fibers spanning multiple fields were acquired at higher magnification and resolution using an HC PL FLUOTAR 20×/0.4 objective in the DAPI channel (excitation and emission filters: 407/375 and 450/20) at 27% illumination intensity, exposure time of 100 ms, and 2.7 μm step size (SVI Nyquist calc., https://svi.nl/Nyquist-Calculator). Image stacks were then stitched in the LAS X software for downstream analysis.

### 3D myonuclear quantification using IMARIS

Raw, merged z stacks of single fibers (Figure 2A) were first converted to the “.ims” format before being imported into IMARIS for further analysis. Prior to myonuclear quantification, images were processed with a background subtraction algorithm (filter width = 10 μm) to enhance myonuclear signal relative to the fiber background (Figure 2B). Myonuclei from multiple single fibers were measured using a line profile within IMARIS, from which the average diameter of a myonucleus was estimated to be 10 μm, resulting in a background subtraction filter width of 10 μm.

**Figure 2:**
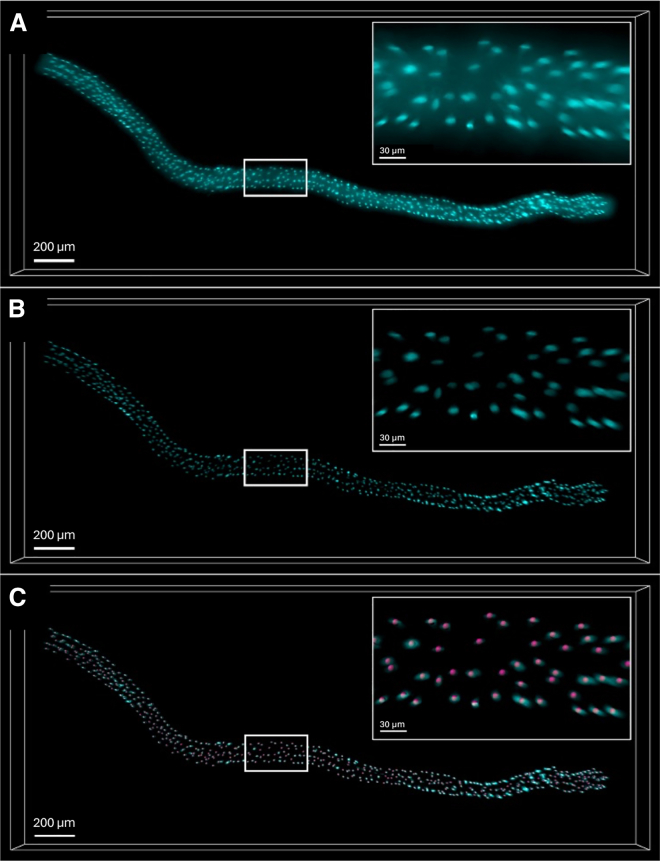
Myonuclear quantification in IMARIS (A) Raw z stack of a single muscle fiber acquired under the DAPI channel (cyan). Scale bars = 200 μm (30 µm, inset). (B) Background-subtracted DAPI channel (filter width = 10 μm). Scale bars = 200 μm (30 µm, inset). (C) Myonuclear quantification on single muscle fiber; Spot (magenta) placed at center of each identified myonucleus (cyan). Scale bars = 200 μm (30 µm, inset).

“IMARIS’ Spots” module was used for accurate myonuclear quantification. First, the “estimated XY diameter” of the feature object was input as 10 μm based on previously described myonuclear measurements. IMARIS’ Spots prediction was then thresholded using “IMARIS’ Quality” parameter to obtain myonuclear counts (Figure 2C). Total counts were manually quality-controlled by including counts of myonuclei not picked up by the “Spots” algorithm due to weak fluorescence signal, as well as manually excluding Spots that were incorrectly placed on aberrant DAPI signal or on nuclei outside of the muscle fiber.

When comparing IMARIS’ total myonuclear counts without manual quality control to counts with manual quality control on 619 fibers, a Bland-Altman analysis resulted in a bias of 3.833% and 95% limits of agreement from −3.479% to 11.14% (Figure 3A). Across the 619 fibers, the average nuclei/mm of muscle tissue with manual correction was 167.4 compared with 160.9 without manual correction. The r value between methods was 0.9910 (Figure 3B) and the average percent error was 4.02 × 10^−3^. IMARIS tended to have lower counts without manual quality control, potentially due to nuclei clustering on top of each other, preventing Spots from accurately segmenting the clustered object as multiple individual myonuclei. Fibers used for this validation were obtained from a subset of 46 biopsies from the 9 subjects in Table 1. As myonuclear number is a primary outcome for our projects, we chose to include this manual quality control.

**Figure 3:**
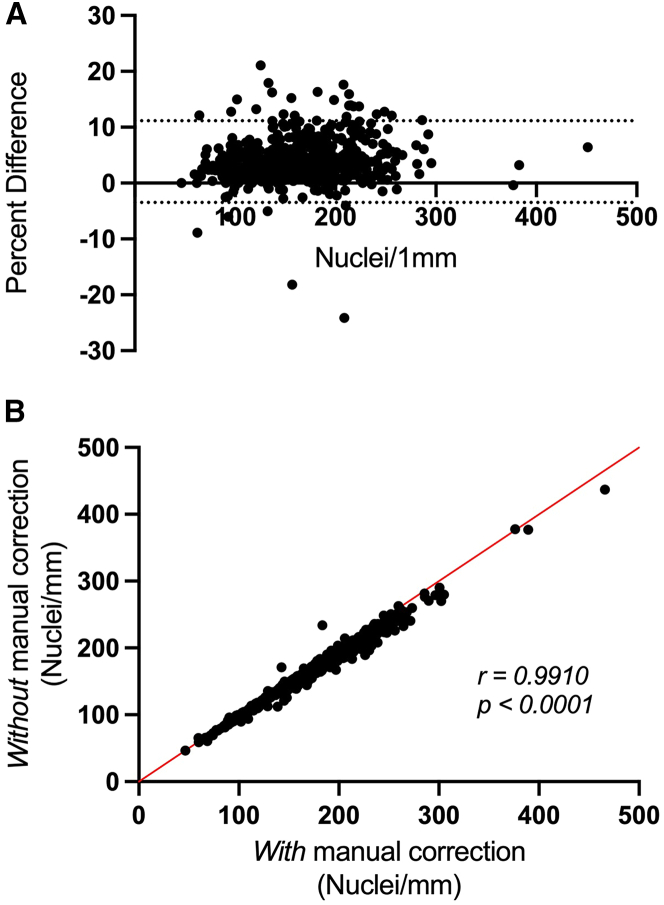
Comparison of nuclei counted per mm of muscle tissue from IMARIS with and without manual quality control (*N* = 619 fibers) (A) Bland-Altman 95% limits of agreement plot comparing IMARIS’ nuclei counts with and without manual correction. (B) Correlation between nuclei/per mm of muscle tissue with and without manual correction; r = 0.9910 and *p* < 0.0001.

To further increase our confidence in the manual quality control for IMARIS analysis, we performed both intra- and inter-rater comparisons. 20 fibers from the original analysis (labeled expert V1) were reanalyzed, first by the same individual who performed the original analysis (labeled expert V2) and by a separate individual trained on IMARIS (labeled novice). The expert had analyzed over 2,000 single fibers, while the novice had analyzed under 100 single fibers. Bland-Altman analysis between expert V1 and expert V2 resulted in a bias of −0.7911% and 95% limits of agreement from −4.370% to 2.787% (Figure 4A). The r value between methods was 0.9996 (Figure 4B), and the interclass correlation coefficient as calculated by Python was 0.9993. Bland-Altman analysis between expert V1 and novice resulted in a bias of −0.8719% and 95% limits of agreement from −5.574% to 3.830% (Figure 4C). The r value between methods was 0.9988 (Figure 4D), and the interclass correlation coefficient as calculated by Python was 0.9986. Fibers used for this validation were obtained from a subset of 2 biopsies from the 9 subjects in Table 1.

**Figure 4:**
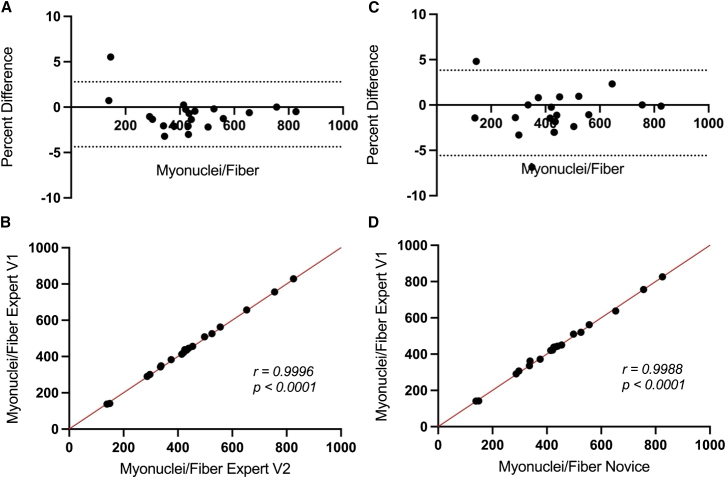
Intra- and inter-rater comparison of IMARIS manual quality control step (*N* = 20 fibers) (A) Bland-Altman 95% limits of agreement plot comparing expert V1 and expert V2. (B) Correlation between expert V1 and expert V2; r = 0.9996 and *p* < 0.0001. (C) Bland-Altman 95% limits of agreement plot comparing expert V1 and novice. (D) Correlation between expert V1 and novice; r = 0.9988 and *p* < 0.0001.

### 3D fiber length measurement

Fiber length is obtained using “IMARIS’ Filaments” module. First, a mask of the entire fiber is created using the “Surface Creation” module. The whole single fiber is segmented on the DAPI channel using a smoothing width of 15 μm and then masked and binarized by setting the pixel value within the segmented surface to 1.00. These masking steps in the Surface Creation module result in a duplicated DAPI channel with a consistent signal throughout the entire single muscle fiber in all three dimensions.

IMARIS uses representative “Seed Points” placed along the fiber as a guide to estimate the path that the “Filament” should follow along the DAPI signal. This method optimizes both the source channel with a smoothed, binarized DAPI signal, as well as optimal Seed Points that are manually placed along the fiber. After indicating the “thinnest diameter” of the object of interest as 100 μm (approximate diameter of a muscle fiber), IMARIS’ default Seed Points are removed. Then, in “Slicer View” with a slice width of 10 μm, new seed points are manually placed at the center of the fiber from end-to-end. This results in a smoothed filament (Figure 5) that follows the center of the muscle fiber. Fiber length is measured by obtaining the filament length in microns. For validation of this method, 88 single muscle fibers were measured manually using 150 μm segments placed along the fiber’s central axis in IMARIS’ Slice View mode. Bland-Altman analysis between manual and IMARIS-derived fiber lengths resulted in a bias of 0.279% and 95% limits of agreement from −2.511% to 3.069% (Figure 6A). Across the 88 fibers tested, the average fiber length was 3,054 μm when measured manually compared to 3,045 μm when measured by IMARIS. The r value between manual and IMARIS-derived fiber lengths was 0.9997 (Figure 6B) and the average percent error was 2.69 × 10^−5^. Fibers used for this validation were obtained from a subset of 10 biopsies from the 9 subjects in Table 1. We chose to go forward with the IMARIS-derived fiber length measurements for our analyses.

**Figure 5:**
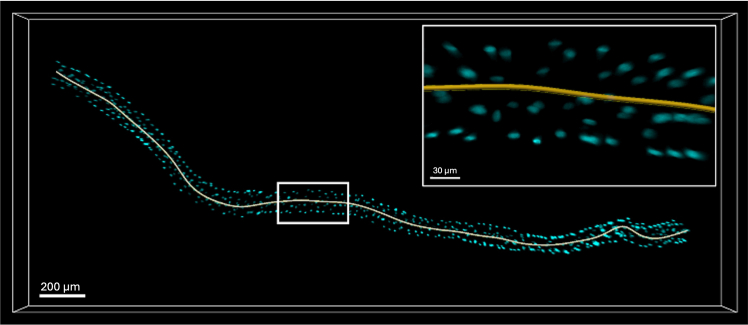
Fiber length measurement in IMARIS Single muscle fiber with a Filament (yellow) placed along the center of the fiber from end-to-end (fiber length = 3,061 μm). Scale bars = 200 μm (30 µm, inset).

**Figure 6:**
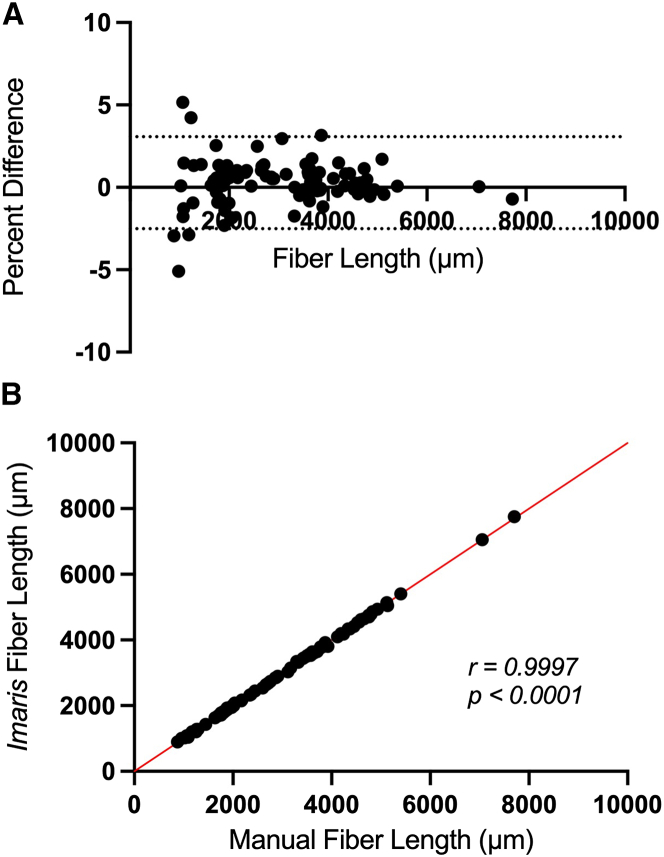
Comparison of fiber length in micrometers from IMARIS and manual methods (*N* = 88 fibers) (A) Bland-Altman 95% limits of agreement plot comparing IMARIS’ fiber length measurement to manual measurement. (B) Correlation between fiber length from IMARIS and manual methods; r = 0.9997 and *p* < 0.0001.

### 2D analysis of myonuclear shape, position, orientation, and co-localization

3D segmentation using 20× widefield z stacked images in IMARIS allowed us to efficiently quantify myonuclear density from a large number of fibers; however, when attempting to 3-dimensionally render myonuclear morphology from these images, *z* axis elongation and excessive light scattering distorted the true shapes of the myonuclei. This distortion, an inherent issue with widefield systems, resulted in nuclei appearing artificially elongated in the *z* dimension.

To glean information on myonuclear shape, position, and orientation, while still utilizing widefield images that can be collected with relative ease, we decided to pivot to a 2D analysis using 2D projections of the original 3D z stacks. This 2D visualization allows us to meaningfully and accurately group myonuclei of similar shapes using aspect ratio—a metric that has been applied by other groups in the field.^10^^,^^15^^,^^16^^,^^17^ Additionally, this approach allows us to capture information about myonuclear orientation and colocalization, all without the added artifacts that the *z* dimension adds to these images.

To do this, we developed a fully automated macro pipeline in FIJI designed to convert each z stack into a 2D standard deviation (STD) projection for efficient 2D morphometric analysis of each myonucleus.^18^ This approach collapses the z stack into a single image that emphasizes variation in intensity across z planes, effectively highlighting myonuclei while reducing the signal of unwanted artifacts produced in the *z* dimension during image acquisition. Importantly, this also allows for robust thresholding and segmentation steps to be applied consistently across images, greatly improving throughput and reproducibility for efficient automated processing of large datasets.

### Overview of the FIJI macro pipeline

The macro was designed to handle batch processing of Leica “.lif” files and automatically parses sample metadata (SubjectID, time point, and leg) to organize outputs. It runs fully automated in FIJI macro language batch mode and includes the following steps:

1. Image import and preprocessing

Each .lif file is opened using Bio-Formats. The macro selects only those series labeled “Merged” for analysis. Prior to image processing, a raw TIFF of the z stack (Figure 7A) is saved downstream z plane identification of objects. A rolling ball background subtraction (radius = 30 pixels, 9.87 μm, the average diameter of a myonucleus) is applied to the z stack to enhance nuclear contrast (Figure 7B).

2. Standard deviation Z projection

**Figure 7:**
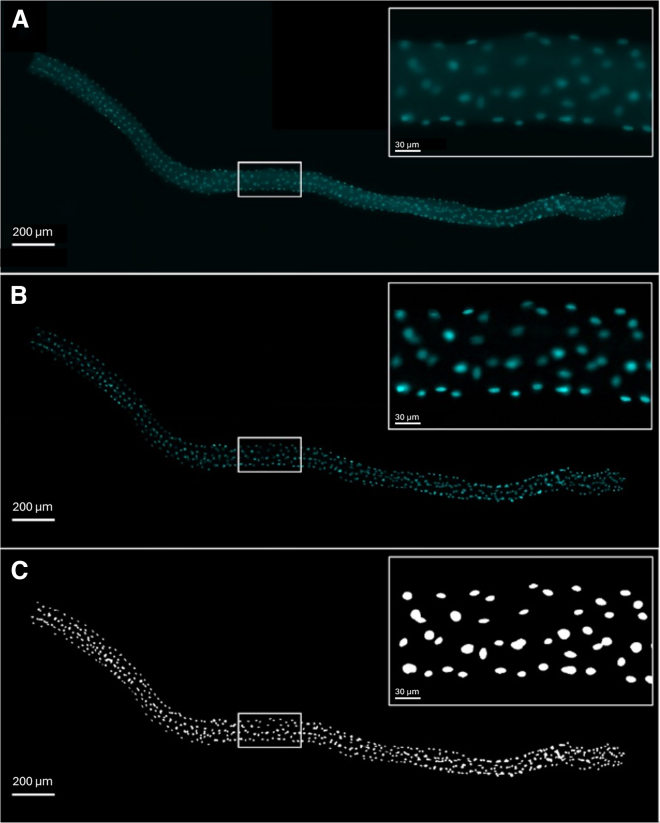
2D nuclei mask generation (A) Single muscle fiber z stack acquired under the DAPI channel (358 nm). Scale bars = 200 μm (30 µm, inset). (B) Background-subtracted DAPI channel (filter width = 10 μm). Scale bars = 200 μm (30 µm, inset). (C) STD z projection of the DAPI channel following binarization with Otsu thresholding + median filter (filter width = 2 px). Scale bars = 200 μm (30 µm, inset).

The macro generates a 2D projection using the “standard deviation” method, which accentuates objects that differ in mean intensity across z slices, which is ideal for identifying myonuclei. A median filter (radius = 1 pixel) is applied to reduce noise before automatic thresholding via Otsu’s method. The image is binarized and converted to an 8-bit mask to ensure compatibility with downstream processing (Figure 7C). These projections are saved under a “STDIP” directory for each sample.

3. Skeletonization workflow

The projected image is duplicated, blurred (Figure 8A), and thresholded again with Otsu (Figure 8B). This step transforms the DAPI mask created in step 2 into a representative mask of the overall fiber shape. FIJI’s Analyze Particles is then used to isolate objects larger than 70,000 pixels^2^ (23,020 μm^2^), thus segmenting out any smaller objects remaining in the image. This mask is converted to 8-bit binary to guarantee compatibility with the Skeletonize function in FIJI (Figure 8C). The resulting 1-pixel-wide skeleton is saved under a “Skel” directory.

4. File structure and organization

**Figure 8:**
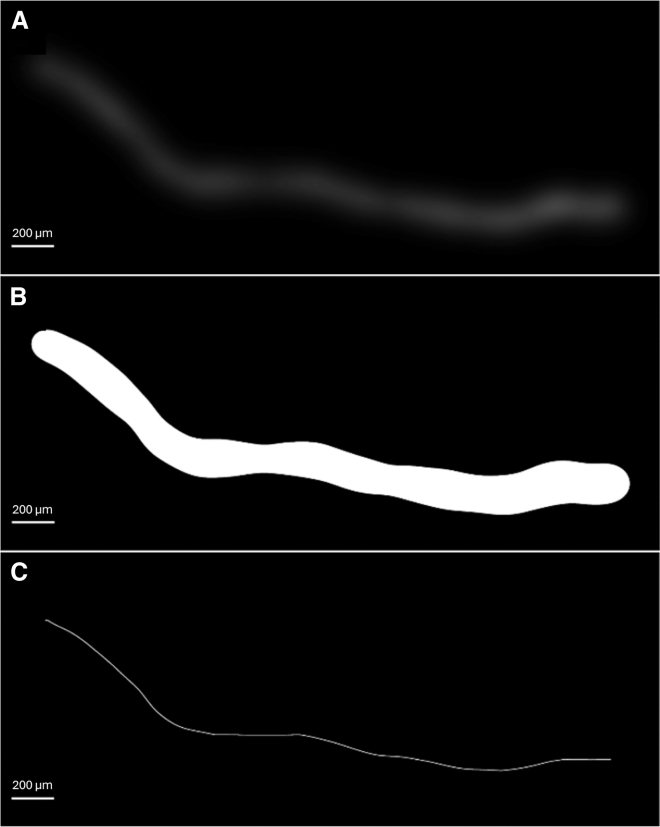
2D fiber skeleton generation (A) Gaussian-blurred nuclei mask (σ = 55 μm). Scale bars, 200 μm. (B) Gaussian-blurred nuclei mask following Otsu thresholding. Scale bars, 200 μm. (C) Skeleton for representation of fiber orientation (original skeleton width = 1 px; image has undergone 6 iterations of dilation for visualization). Scale bars, 200 μm.

Outputs are organized automatically by subject, time point, and leg in nested directories.

### Python-based myonuclear analysis

We developed a Python-based image analysis pipeline to quantify nuclear morphology, spatial position, orientation, and clustering within individual skeletal muscle fibers. The project was initially conceived as a hybrid CellProfiler-Python workflow, but the final implementation achieved greater flexibility and analytical control through a fully Python-based design. The workflow processes each fiber’s input images—binary nuclear masks (STDIP), corresponding fiber skeletons, and z stacked fluorescence images—to extract per nucleus morphometric and spatial features.

The pipeline was designed to (1) ensure reproducible per-fiber processing, (2) maintain compatibility with existing file-naming and organizational conventions used in the Dreyer Lab, (3) enable flexible parameter tuning for microscope pixel scaling, depth spacing, and noise filtering, and (4) generate transparent outputs suitable for quality control and further statistical aggregation.

#### Input structure and parsing

Each fiber is represented by three co-registered images stored within standardized subdirectories:

• *STDIP/*—binary standard-deviation projection image marking nuclear masks;
• *Skel/*—2D representative fiber skeleton image; and
• *TIFs/*—z stacked 3D image with dimensions (*Z*, *Y*, *X*).

The parser is robust to naming variants and missing files.

#### Parameterization

All adjustable constants—such as pixel-to-micron conversion, *z* axis scaling, area filtering limits, and clustering radius—are stored in a “Params” dataclass. This allows all analysis steps to reference a single structured parameter object and supports command-line overrides; encapsulating parameters in a dataclass makes the pipeline reproducible and portable across imaging conditions and magnifications.

#### Image loading and preprocessing

Utility functions handle file input and preparation:

• _imread() loads TIF/TIFF files using scikit-image.
• _to_bool() ensures binary STDIP masks are converted to Boolean arrays.
• _label_mask() labels nuclei in the binary image, optionally removing objects smaller than a given minimum area (min_area_px = 200, 65.831 μm^2^).

#### Nuclear morphology measurements

The function measure_nuclei_from_binary() computes morphometric descriptors for each labeled nucleus of the STDIP image: area, perimeter, centroid coordinates, major/minor axis lengths, and orientation. Derived quantities include the following:

• Aspect ratio = minor/major axis length. A value of 1 indicates a perfect circle, and values approaching 0 indicate an ellipse.
• Shape class: to categorize nuclei by geometric eccentricity, we established discrete boundaries: elongated (<0.55), intermediate (0.55–0.80), and circular (>0.80) (Figure 9). These thresholds were derived from the empirical frequency distribution of the aspect ratios within our dataset, identifying natural clustering to distinguish compressed or obliquely sectioned nuclei from those with high circularity. This threshold should be empirically determined based on research context/model.

**Figure 9:**
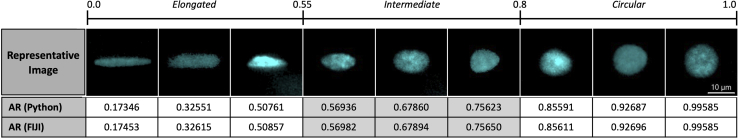
Shape classes Representative images of nuclei ranging from the most elongated to the most circular, illustrating the three shape classes and their corresponding aspect ratio thresholds. Aspect ratio is shown as calculated by Python and as calculated by FIJI for each representative nucleus. Images were taken from DAPI visualization in IMARIS, and differing intensities are a result of how close or far a nucleus was from the objective during imaging. Scale bars, 10 μm.
• Orientation (deg) on 0°–180° scale.

All measurements are then converted from pixel to micrometer units (pixel_size_xy_μm = 0.3291532 for 20× objective), specific to imaging protocol. Annotated mask overlays are also generated for quality control.

For validation of Python-derived aspect ratios, 275 myonuclei were measured both by FIJI and by the Python script. Bland-Altman analysis between FIJI and Python-derived aspect ratios resulted in a bias of 0.0818% and 95% limits of agreement from −0.0815% to 0.245% (Figure 10A). The r value between FIJI and Python-derived aspect ratios was 1.000 (Figure 10B) and the average percent error was 0.082%. Nuclei used for this validation were obtained from a subset of 1 biopsy from the 9 subjects in Table 1. Because aspect ratio is mathematically defined, and because both FIJI and Python compute aspect ratio using the same formula, the two methods have essentially identical outputs (barring some rounding differences).

**Figure 10:**
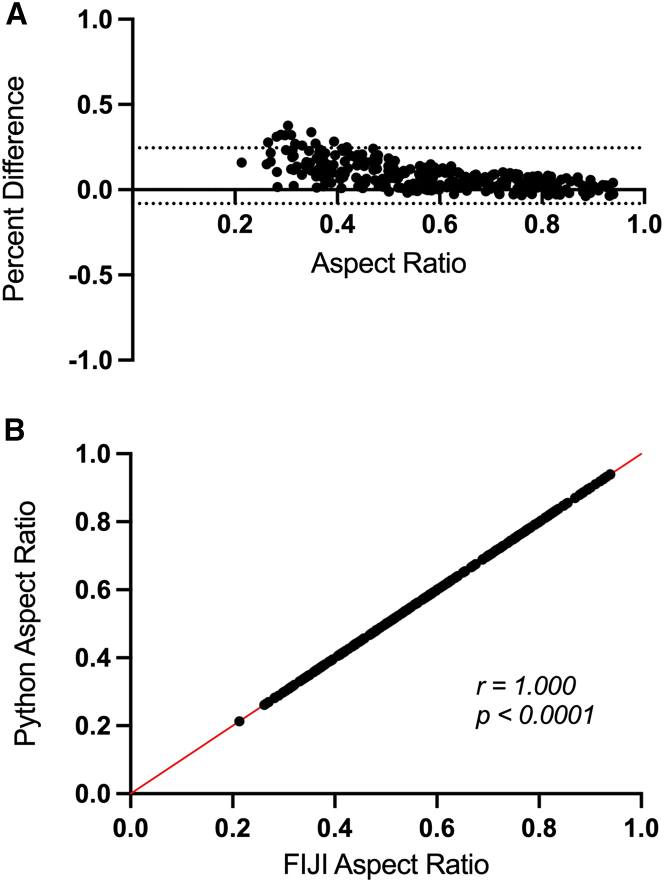
Comparison of aspect ratio from FIJI and Python methods (*N* = 275 nuclei) (A) Bland-Altman 95% limits of agreement plot comparing FIJI-derived aspect ratios with Python-derived aspect ratios. (B) Correlation between aspect ratio from FIJI and Python; r = 1.000 and *p* < 0.0001.

#### Z position assignment and z consistency filtering

Because widefield imaging introduced substantial *z* axis elongation artifacts, *z* dimensions from microscope metadata could not be used for reliable 3D measurements or spatial clustering. To mitigate this distortion, the function “compute_mean_z_for_labels()” estimates the effective Z position directly from the intensity data. For each pixel, the index of maximal fluorescence intensity (z-argmax) is identified to produce a per-pixel map of maximal signal depth. For each nucleus, the mean Z position is accepted only when the within-object standard deviation (Z-STD) of these indices satisfies a dynamic consistency filter.

To distinguish valid biological structures from vertically overlapping artifacts or optical “flare,” the pipeline applies the following logic:

• *Planar nuclei*: objects with larger XY footprints are restricted to a strict Z-STD ≤2.0 to ensure they represent a single focal plane.
• *Vertical nuclei:* to prevent the disproportionate exclusion of nuclei oriented perpendicular to the imaging plane (e.g., at the lateral fiber boundaries), a more lenient threshold is applied to objects with small XY areas (<500 pixels, 164.5 μm^2^). This accounts for the natural axial span of a “vertical” nucleus while still rejecting non-biological projection artifacts.

This dual-threshold approach yields a robust *Mean_Z_um* measure for all included nuclei and improves downstream 3D spatial analysis. Both included and excluded nuclei are retained in separate CSV files, ensuring full traceability of the filtering process.

#### Relative myonuclear orientation

While the preceding morphometric measurements (Section nuclear morphology measurements) describe nuclear orientation relative to the global *x*-*y* image axes, this does not capture the true alignment of nuclei with respect to individual muscle fibers, whose trajectories may curve or vary across the tissue section. To resolve this, the function *skeleton_angles_near_nuclei()* estimates the dominant local direction of each myofiber in the vicinity of every nucleus.

For each nuclear centroid, the algorithm identifies all skeleton pixels within a fixed radius and applies principal-component analysis (PCA) to their (*x*, *y*) coordinates. The first principal component corresponds to the dominant local axis of the fiber segment. Nuclear orientation, originally measured relative to the image *x* axis, is then compared with this local fiber axis to compute a relative alignment angle (Figure 11).

**Figure 11:**
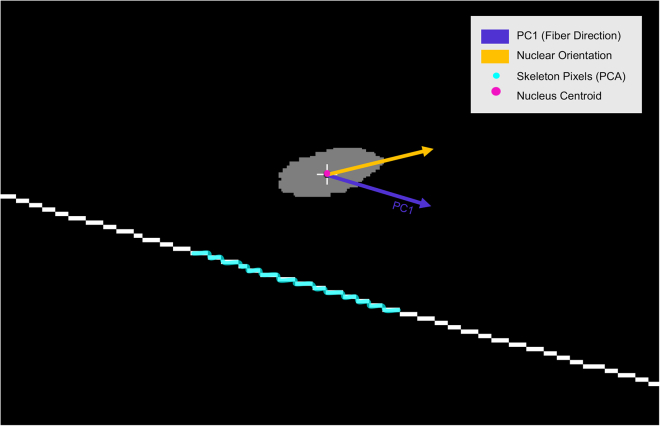
Raw Python generated image of principal-component analysis of local fiber direction Local fiber direction is estimated by applying PCA to the coordinates of skeletonized fiber pixels within a fixed radius around each nucleus; the first principal component (PC1) indicates the dominant fiber direction.

This relative angle is expressed on a 0°–90° scale, where 0° denotes perfect alignment of the nucleus with the fiber axis and 90° indicates perpendicular orientation. This approach provides a curvature-aware measure of nuclear alignment that reflects local myofiber structure rather than global image geometry (Figure 12).

**Figure 12:**
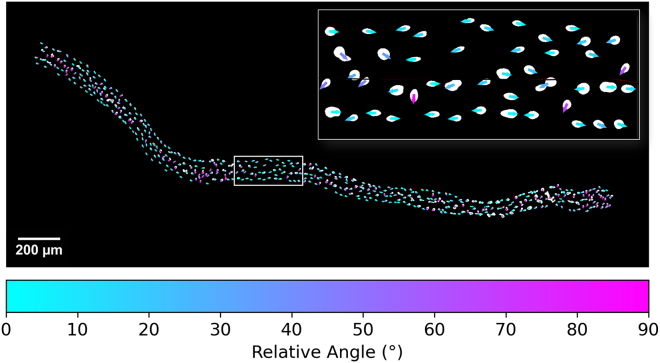
Raw Python generated image of relative orientation angles of myonuclei along a single myofiber Each nucleus is overlaid with a colored arrow representing the orientation of the long axis of the nucleus to the long axis of the myofiber. A cyan arrow indicates parallel alignment, and a magenta arrow indicates perpendicular alignment to the long axis of the myofiber. Scale bars, 200 μm.

#### Distance to fiber skeleton

To quantify nuclear positioning relative to the fiber core, *compute_distance_to_skeleton()* calculates the Euclidean distance from each nucleus centroid to the nearest skeleton pixel. This produces a *DistanceToSkel* variable (μm) for every nucleus. This metric enables downstream classification of “central” nuclei and supports nuclear chain analysis.

#### 3D clustering of nuclei

To identify spatial clusters of myonuclei within individual muscle fibers, we applied the Density-Based Spatial Clustering of Applications with Noise (DBSCAN) algorithm via the *sci-kit learn* library. DBSCAN was chosen for its ability to identify clusters of arbitrary shape based on local point density while effectively segregating outliers. Clustering was performed using 3-dimensional myonuclear centroid coordinates (position X, position Y, and position Z).

The algorithm was parameterized with a *min_samples* value of 2 (defining a cluster as at least two nuclei) and an *eps* value of 20 μm. As 10 μm is the average diameter of a single nucleus, an eps value of 20 μm corresponds to the diameters of two nuclei. Therefore, a cluster is defined as two or more nuclei that are not more than 20 μm (two nuclei) apart from one another. This parameter can be adjusted based on how the user would like to define clustering in their model. For example, increasing the eps by 5 μm will widen the search range, resulting in fewer clusters with more nuclei in each. Similarly, decreasing the eps by 5 μm will decrease the search range, resulting in more clusters with fewer nuclei in each.

Cluster labels assigned by DBSCAN were then added to the dataset with unclustered nuclei (noise) labeled as −1 (Figure 13).

**Figure 13:**
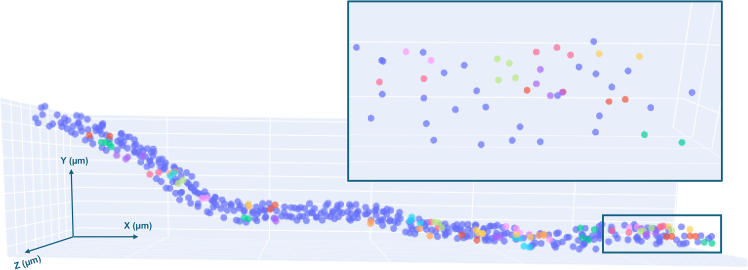
Raw Python generated image of 3D myonuclear spatial analysis with DBSCAN Cluster labels assigned by DBSCAN visualized using Plotly in a scatterplot. Each myonucleus is colored according to its cluster identity; nuclei not assigned to any cluster are labeled as −1 and displayed in the plot in gray.

#### Nearest-neighbor analysis

To further characterize the local nuclear microenvironment, the pipeline computes 3- and 5-nearest-neighbor (NN3/NN5) features for each included nucleus using 3D Euclidean distances. Extracted contextual features include the following:

• *NNk_MeanDist_um* – local nuclear packing density
• *NNk_MeanAspectRatio* – neighborhood shape profile
• *NNk_Frac_Circular/elongated/intermediate* – shape class composition
• *NNk_Frac_Parallel*/perpendicular/IntermediateOrientation – orientation coherence within the microenvironment
• *NNk_MeanRelAngle_deg* – neighbor alignment relative to local fiber axis

These metrics quantify nuclear neighborhood structure and support analysis of “myonuclear footprint” organization.

#### Fiber diameter profiling

The function *compute_fiber_width_profile()* samples diameters along the skeleton at fixed intervals (default 100 μm). Diameters are estimated by projecting included nuclei positions perpendicular to the local axis and measuring left–right separation. This yields a diameter profile and summary statistics (mean, min, max, SD) to inform downstream volume analyses.

For validation of fiber diameter measurement, the diameters of 49 single muscle fibers were measured manually in IMARIS at 100 μm intervals along the fiber; the average of these measurements was used as the mean diameter for the fiber. Bland-Altman analysis between manual and Python-derived fiber diameters resulted in a bias of −7.493% and 95% limits of agreement from −19.26% to 4.273% (Figure 14A). The r value between manual and Python-derived fiber lengths was 0.9411 (Figure 14B) and the average percent error was 8.57%. Fibers used for this validation were obtained from a subset of 3 biopsies from the 9 subjects in Table 1.

**Figure 14:**
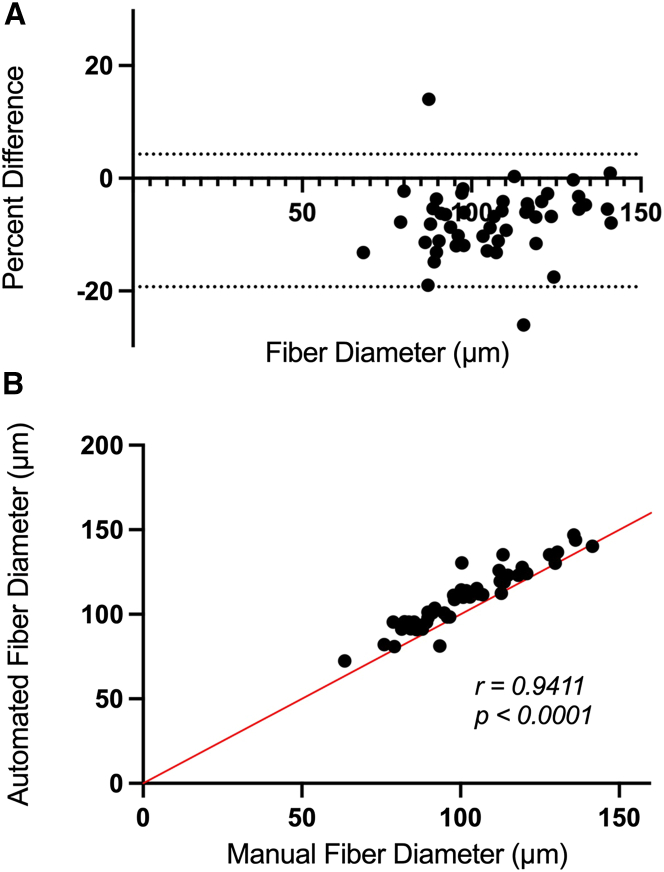
Comparison of fiber diameter in micrometer between manual and Python methods (*N* = 49 fibers) (A) Bland-Altman 95% limits of agreement plot comparing manual fiber diameter measurement to Python measurement. (B) Correlation between fiber diameter from manual and Python; r = 0.9411 and *p* < 0.0001.

#### Myonuclear domain volume

The pipeline also computes a volume-based myonuclear domain for each fiber:

• *FiberVolume* (μm^3^) = π(MeanDiameter/2)^2^ IMARISFiberLength
• *DomainVolume_um3_Python* = FiberVolume/PythonMyonuclei
• *DomainVolume_um3_IMARIS* = FiberVolume/IMARISMyonuclei (if available)

This provides a volumetric estimate of the cytoplasmic territory associated with each myonucleus.

For validation of fiber volume measurement, the volumes of 49 single muscle fibers were calculated from the manual diameter measurements and the length measurement for each fiber using the formula for the volume of a cylinder (π∗r2∗h). Bland-Altman analysis between manual volume and automated volume resulted in a bias of −14.93% and 95% limits of agreement from −38.25% to 8.391% (Figure 15A). The r value between manual and Python-derived fiber volumes was 0.9921 (Figure 15B), and the average percent error was −13.31%. Fibers used for this validation were obtained from a subset of 3 biopsies from the 9 subjects in Table 1.

**Figure 15:**
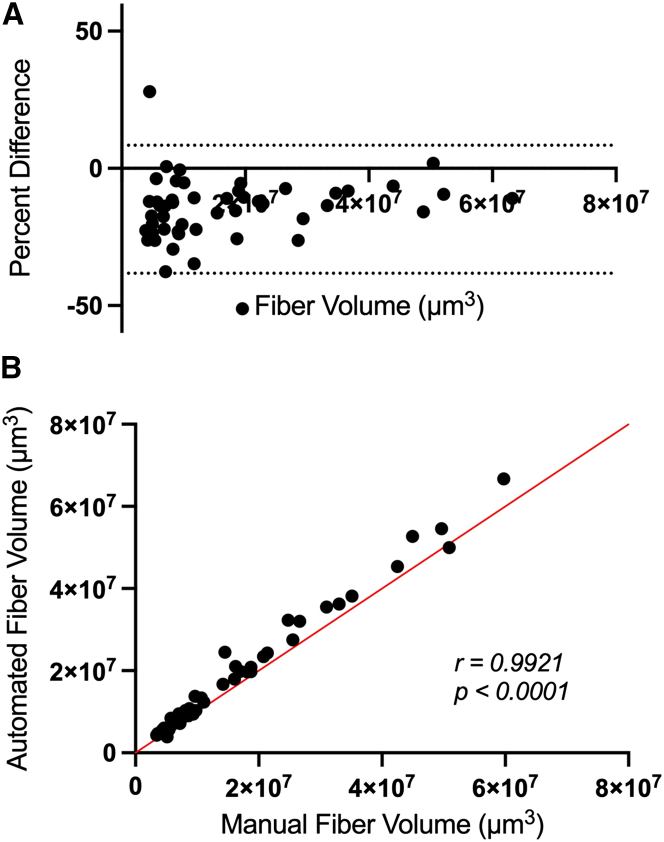
Comparison of fiber volume in cubic micrometer between manual and Python methods (*N* = 49 fibers) (A) Bland-Altman 95% limits of agreement plot comparing manual fiber volume measurement to Python measurement. (B) Correlation between fiber volume from manual and Python; r = 0.9921 and *p* < 0.0001.

For validation of myonuclear domain measurement, the myonuclear domains of 49 single muscle fibers were calculated from the manual volume measurements and the total myonuclei for each fiber. Bland-Altman analysis between manual domain and automated domain resulted in a bias of −14.93% and 95% limits of agreement from −38.25% to 8.391% (Figure 16A). The r value between manual and Python-derived fiber volumes was 0.9592 (Figure 16B) and the average percent error was 17.02%. Fibers used for this validation were obtained from a subset of 3 biopsies from the 9 subjects in Table 1.

**Figure 16:**
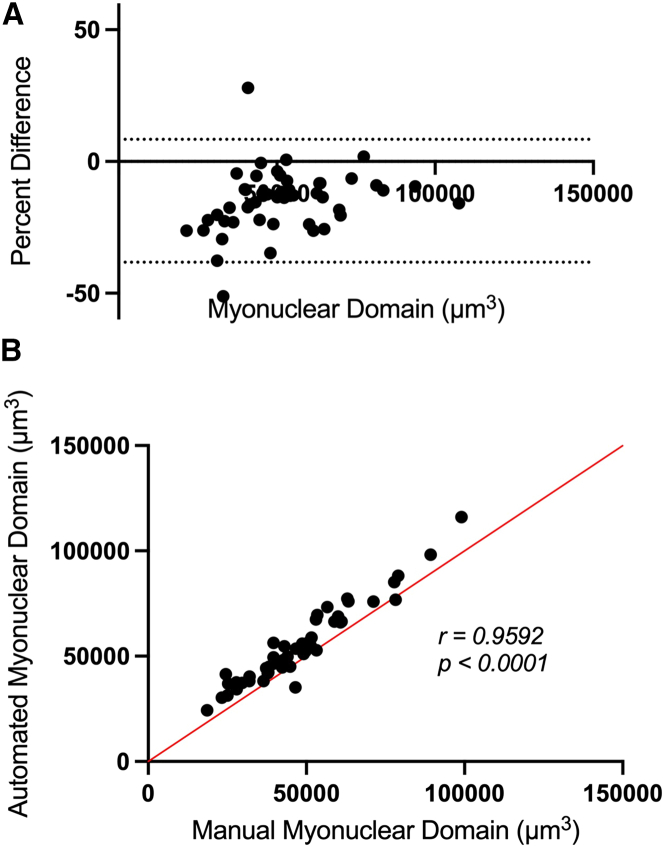
Comparison of myonuclear domain in cubic micrometer between manual and Python methods (*N* = 49 fibers) (A) Bland-Altman 95% limits of agreement plot comparing manual myonuclear domain measurement to Python measurement. (B) Correlation between myonuclear domain from manual and Python; r = 0.9592 and *p* < 0.0001.

#### Biopsy-level summaries

For each fiber, derived results are merged with IMARIS master file metrics (fiber length and nuclei counts), if available. Additional biopsy-level variables are computed:

• Nuclear counts (PythonMyonuclei, IMARISMyonuclei)
• Diameter statistics and FiberVolume
• Myonuclear domain volume (Python/IMARIS)
• Neighbor microenvironment metrics (fiber-level means of NN3 features)
• Shape and orientation distributions
• Cluster# (3D DBSCAN clusters)
• Nuclear chain and central-nuclei counts

#### Batch orchestration and outputs

Helper functions automate batch processing across subjects, timepoints, and legs. Each subject receives a workbook *(<Subject>_results.xlsx)* with one sheet per timepoint, preserving column order and preventing Excel auto formatting of fiber IDs. Each fiber output include the following:

• *nuclei_results.csv* (included nuclei)
• *excluded_nuclei.csv* (excluded objects)
• *fiber_width_profile.csv* (diameter sampling)
• *overlay.png* (annotated mask image)

Biopsy level summaries integrate Python metrics with outputs, providing a unified dataset for downstream statistical analysis.

This workflow integrates morphometric, orientational, and 3D spatial analyses of single-fiber nuclei into a unified Python framework. By combining region-property extraction, Z position inference, curvature-aware orientation estimation, diameter profiling, nearest-neighbor microenvironment analysis, and unsupervised clustering, the pipeline yields a rich quantitative description of myonuclear organization without the need for proprietary software. The modular design enables adaptation to a range of staining protocols and imaging systems while producing fully auditable outputs for downstream statistical modeling and biological interpretation.

A summary of average values for primary outputs is provided in Table 2.

**Table 2:** Data summary

	Type I	Type II
Number of fibers	89	126
Myonuclei (mm)^a^	168.9 ± 57.77	161.9 ± 44.37
Diameter (μm)	116.88 ± 31.1	115.9 ± 33.73
Fiber volume (μm^3^)	3.08 × 10^7^ ± 2.84 × 10^7^	2.89 × 10^7^ ± 3.17 × 10^7^
Myonuclear domain (μm^3^)	7.20 × 10^4^ ± 4.70 × 10^4^	6.85 × 10^4^ ± 3.29 × 10^4^
Nuclei excluded by Python (%)	37.32 ± 16.84	39.38 ± 14.12
Elongated (%)	31.51 ± 11.22	60.16 ± 19.46∗
Intermediate (%)	50.76 ± 9.07∗	38.34 ± 13.08
Circular (%)	18.79 ± 3.84∗	9.79 ± 6.79
Parallel orientation (%)	10.97 ± 3.88	11.36 ± 4.61
Intermediate orientation (%)	79.03 ± 6.23	76.35 ± 5.86
Perpendicular orientation (%)	11.10 ± 5.58	9.39 ± 8.11
Clusters (mm)	11.47 ± 6.17∗	8.17 ± 4.63
Nearest neighbor distance (μm)	37.3 ± 1.34	38.94 ± 3.61

Fibers used for this validation were obtained from a subset of 16 biopsies from the 9 subjects in Table 1. Data were analyzed using unpaired *t* test. Data are presented as mean ± SD.

∗*p* < 0.0001 between fiber types.

a Myonuclei quantified by IMARIS.

## Discussion

In this study, we developed and validated a semi-automated pipeline analysis method to quantify myonuclei number and structural features along the length of individual single muscle fibers from biopsy. Our approach allows for reproducible, high-resolution measurement of myonuclear number, morphology, clustering, and orientation from immunohistochemical images. By integrating myonuclear density metrics with spatial orientation of similarly shaped myonuclei, our method provides a foundation for examining how nuclear organization relates to multinucleated muscle cell plasticity.

Although several automated pipelines exist for analyzing muscle ultrastructure, most are designed for tissue cross-sections, where sampling many fibers simultaneously increases statistical confidence at the tissue level. In contrast, our motivation was to maximize individual muscle cell-specific power to quantify myonuclear accretion and local and longitudinal organization. This required sampling along the length of single fibers to capture the true spatial relationships of nuclei that cannot be inferred from cross-sectional preparations.

Our first aim was to achieve accurate quantification of myonuclear number along the length of individual single fibers, enabling precise density metrics such as myonuclei per millimeter and myonuclei per volume. By combining FIJI-based preprocessing with Python analysis and integrating fiber-length measurements from IMARIS, our pipeline generates reproducible nuclear counts that ensure reproducible quantifiable data. This approach provides confidence in density estimates that are difficult to achieve with manual methods. Compared to cross-sectional imaging pipelines, which maximize statistical power by sampling many fibers simultaneously, our longitudinal approach prioritizes fidelity in capturing nuclear accretion and local organization along the fiber axis.

The second aim was to develop a scalable method for representing merged DAPI z stacks into a standardized format suited for batch processing. Because widefield z stacks generate large, computationally demanding datasets, our FIJI macro reduces file size by generating standardized 2D projections while preserving nuclear structural information. This design allows efficient downstream analysis and maintains compatibility with existing file-naming conventions.

The third aim was to establish a reproducible framework for quantifying nuclear morphology, orientation, clustering, and spatial relationships from 2D z projections. The Python implementation extracts morphometric parameters from binary nuclear masks—including area, perimeter, centroid location, axis lengths, aspect ratio, and shape class—producing standardized metrics for comparisons across fibers or conditions. Orientation is calculated relative to the local fiber axis, determined via PCA of nearby skeleton pixels that define trajectory with nuclear orientation, yielding alignment angles (0°–90°) that account for fiber curvature and not global image axes.

Spatial organization is characterized through DBSCAN clustering of 3D centroid coordinates, which identifies discrete nuclear clusters and labels noise nuclei, and through centroid-to-skeleton distances that enable classification of central nuclei and central nuclear chain formations. Fiber organization is further interrogated with nearest-neighbor statistics, which utilizes local density patterns to reveal whether the nuclei tend toward random, clustered, or ordered distributions.

Together, these measures generate a reproducible “fingerprint” for each muscle fiber, integrating morphometric, orientational, and spatial descriptors into a unified profile. This fingerprinting framework allows for longitudinal and comparative studies, where distinct nuclear arrangements such as linear nuclear chain formations may be correlated with biological variables including age, disuse, or exercise status. Compared with existing tools—such as CellProfiler, which provides accessible workflows for segmentation and basic morphometry—IMARIS and Python together provide accurate quantification as well as highly modular parameters that can be extensively adjusted to the user’s preferences. Our pipeline emphasizes transparency, reproducibility, and scalability for widefield datasets.

### Limitations of the study

Limitations include reliance on widefield microscopy, which prevents true 3D morphological assessment (although raw nuclear counts are still performed in 3D for highest confidence). Confocal microscopy could be used for 3D morphological analysis, but in our experience, the necessary imaging parameters on a Nikon SoRa spinning disc confocal microscope would increase imaging time by approximately 40× compared with the widefield microscope used here. Specifically, the Nikon SoRa spinning disc requires a zstep size of 0.4 μm and the use of a 40× water immersion objective, taking about 4 min to render 200 μm of muscle fiber. Conversely, the widefield microscope uses a z step size of 2.7 μm and a 20× air objective, allowing us to image up to 8,000 μm of tissue in ∼4 min. Broadly speaking, this translates to 4 min versus 160 min to image an 8,000-μm-long single fiber, and due to this time constraint, we chose to use widefield microscopy for this method. To overcome the widefield distortion of nuclear morphology in 3D, we compress the z stacked images into a 2D standard deviation projection, allowing us to gather accurate 2D morphological data. A limitation of this approach is that nuclei on the same z plane are sometimes merged into one object, which is then excluded from further morphological analysis by Python as it no longer represents a single true myonucleus. However, our method offers a practical balance between fidelity, scalability, and computational efficiency—making longitudinal single-fiber nuclear analysis feasible across large cohorts.

Other limitations are that the DAPI stain is used as an approximation to measure fiber diameter, and that fiber volume is calculated as the volume of a cylinder from the diameter and length measurements. Myonuclear domain is calculated from the derived fiber volume measurements. Using a stain that is a true boundary marker, such as phalloidin, may improve fiber diameter measurements. The limitation of fiber volume could be resolved by confocal imaging, but the same challenges presented earlier remain.

While the main outputs (myonuclear number, fiber length, aspect ratio, fiber diameter, fiber volume, and myonuclear domain) are validated against alternative methods, parameters such as orientation angle, clustering, and nearest neighbor metrics are difficult to validate with confidence. This is because they are defined by the Python script, measured mathematically, or based on the DBSCAN algorithm, making any manual analysis of these parameters much less accurate than what the Python script can detect.

Additionally, this methods paper should be empirically tested by users to determine applicability in other models. We expect that if similar tissue-processing and imaging parameters are used, this pipeline can be appropriately adjusted to accommodate other models.

In conclusion, we present a semi-automated pipeline that (1) quantifies myonuclear number in 3D along single muscle fibers; (2) classifies nuclei into morphological categories (elongated, intermediate, circular); (3) quantifies the number of similarly shaped nuclei within a defined spatial radius; and (4) determines nuclear orientation relative to the local fiber axis. To the best of our knowledge, this is the first integrated pipeline to apply widefield microscopy—common in most laboratories—to longitudinal single-fiber nuclear analysis. Designed to process large numbers of composite images, this method yields rich datasets describing myonuclear distribution, morphology, and spatial relationships along the fiber. This method has potential for broad application in muscle biology and other fields requiring quantitative analysis of subcellular spatial organization.

## Resource availability

### Lead contact

Requests for further information and resources should be directed to and will be fulfilled by the lead contact, Hans C. Dreyer (hcdreyer@uoregon.edu).

### Materials availability

This study did not generate new reagents.

### Data and code availability

• All data and materials are available in the manuscript and supplemental protocol.
• Sample data are available from https://datadryad.org/dataset/doi:10.5061/dryad.ht76hdrwn.
• All original code is available from https://github.com/DreyerLabUO/23Wu-SingleFiber.

## Acknowledgments

We would like to acknowledge the Genomics & Cell Characterization Core Facility at the 10.13039/100011348University of Oregon. We thank members of the Dreyer Lab for comments and suggestions while testing our pipeline and drafting this manuscript. Graphical Abstract was created using BioRender available from https://BioRender.com/aiz7p7i. This work was supported by the Wu Tsai Human Performance Alliance and the Joe and Clara Tsai Foundation.

## Author contributions

Conceptualization, E.T.S., A.S., and H.C.D.; investigation, E.T.S., H.G.M., M.L., A.S., and H.C.D.; data curation, E.T.S., H.G.M., and M.L.; pipeline methodology/software, E.T.S., H.G.M., M.L., A.S., A.C.F., J.S., and H.C.D.; validation, E.T.S. and H.G.M.; formal analysis, E.T.S., H.G.M., M.L., and H.C.D.; visualization, E.T.S., H.G.M., and M.L.; project administration, H.C.D.; funding acquisition, H.C.D.; writing – original draft, E.T.S., H.G.M., M.L., and H.C.D.; writing – review and editing, E.T.S., H.G.M., M.L., A.C.F., J.S., and H.C.D.

## Declaration of interests

The authors declare no competing interests.

## STAR★Methods

### Key resources table

**Table:** 

REAGENT or RESOURCE	SOURCE	IDENTIFIER
**Antibodies**		

Mouse monoclonal anti-Myosin (Skeletal, Slow)	Sigma-Aldrich	Cat. No. M8421; RRID:AB_477248
Alexa Fluor 555 goat anti-mouse IgG1	Invitrogen	Cat. No. A-21127; RRID:AB_2535769
DAPI Hydrochloride	Invitrogen	Cat. No. D1306

**Biological samples**		

Human muscle biopsy tissue	This paper	IRB#: STUDY00000938

**Chemicals, peptides, and recombinant proteins**		

Vectashield Mounting Medium	Vector Laboratories	Cat. No. H-1000
Paraformaldehyde	Electron Microscopy Sciences	Cat. No. 15710
Sodium Hydroxide (NaOH)	Fischer chemical	Cat. No. SS254-4
Triton-X100	Alfa Aesar	Cat. No. A16046
Phosphate buffered saline	SigmaAldrich	Cat. No. P4417-100TAB

**Deposited data**		

Sample data in GitHub hosted on Dryad data repository for public access	This paper	Dryad:https://doi.org/10.5061/dryad.ht76hdrwn
Original automated Python pipelines for single-muscle-fiber image analysis from the 23_Wu dataset, including fiber width measurement, nuclear orientation, segmentation steps, and batch processing workflows.	This paper	Zenodo:https://doi.org/10.5281/zenodo.18395537
		GitHub:https://github.com/DreyerLabUO/23Wu-SingleFiber

**Software and algorithms**		

ImageJ/FIJI	Schindelin et al.^18^	https://imagej.net/software/fiji/
Python 3.8	Python Software Foundation	https://www.python.org
CellProfiler 4.2.8	Broad Institute	https://cellprofiler.org/citations
IMARIS 11.0.1	Oxford Instruments, Abington, UK	https://imaris.oxinst.com/contact/
SVI Nyquist calc	N/A	https://svi.nl/Nyquist-Calculator
GraphPad Prism 10.4.2 for macOS	GraphPad Prism	www.graphpad.com

### Experimental model and study participant details

#### Human subjects

Single fiber samples isolated from biopsy samples collected from nine human subjects to validate our methods. The study was approved by the University of Oregon Institutional Review Board (STUDY00000938) and was conducted in accordance with Declaration of Helsinki ethical principles. All subjects gave informed written consent prior to study participation.

### Method details

#### Biopsies

Skeletal muscle biopsies samples were obtained using a Bergström needle with 5mm internal diameter with manual suction. Each biopsy yielded approximately 300 mg of tissue (range ∼100—500 mg) with the fiber bundles approximating 2 to 7 mm in length. Our goal for single fiber analysis is to obtain a minimum of 20 individual muscle cells for analysis per biopsy.

#### Single fiber isolation

Immediately following biopsy collection, tissue sections with the longest fiber bundles are teased apart and placed in a 0.6 ml Eppendorf tube containing 500 μl 4% paraformaldehyde (PFA) for 48 hours at 4°C. Fixed fiber bundles are then placed in a 0.6 ml Eppendorf tube containing PBS and kept in the dark at 4°C until processing.

To begin tissue processing, the biopsy sample is placed into a 3 cm diameter glass dish containing a small amount of PBS to prevent drying out. Under a benchtop stereomicroscope, the fixed sample is teased apart with forceps into smaller bundles (final size being ≤ 30 fibers), with care taken to avoid damaging or tearing the fibers. Once separated, the next step is to remove all extracellular components from the sample to further isolate the single fibers. To achieve this, the tissue bundles are transferred into a 1.5 ml Eppendorf tube containing 750 μl of 40% NaOH, then placed on a tube rotator at moderate speed for 2 hours to facilitate digestion of the connective tissue between the fibers. Following digestion, NaOH is removed by filtering the bundle through a 40 μm mesh strainer and repeatedly washed with 1x PBS (10–12 total). Fibers are then collected from the strainer using forceps and transferred into a 0.6 ml Eppendorf tube containing 300 μl 1x PBS until further processing.

Following isolation, fibers are permeabilized in a 1.5 ml Eppendorf tube containing 750 μl 1% Triton-X100 for 10 minutes on the rotor wheel. Permeabilized fibers are then centrifuged at 13,000g for 3 minutes to pellet fibers. After centrifugation, supernatant is removed, and fibers are incubated in primary antibody mouse anti-slow myosin (cat. no. M8421, Sigma-Aldrich, Darmstadt, Germany), 1:200 in PBS for 90 minutes at room temperature. Following incubation in the primary antibody, fibers are washed three times for three minutes in 1% Triton-X100 and centrifuged (13,000g for 3 minutes to pellet fibers). Fibers are then transferred to a 1.5 ml Eppendorf tube containing the secondary antibody Alexa Fluor 555 goat anti-mouse IgG1 (cat. no. A-21127, Invitrogen, Carlsbad, CA, USA), 2:500 in 250 μl PBS and 250 μl 1% Triton-X100 for 1 hour at room temperature. Following incubation in the secondary antibody, fibers are again washed three times for three minutes in 1% Triton-X100, with centrifugation. Fibers are then placed in 100 μl of PBS with 1 μl of 100 μM DAPI (DAPI Hydrochloride, cat. no. D1306, Invitrogen, Carlsbad, CA, USA) for 5 minutes.

Following staining, fibers are transferred to glass microscope slides and carefully separated with forceps to avoid overlap. Vectashield Mounting Medium (H-1000, Vector Laboratories, Newark, CA, USA) is applied to maintain separation and prevent fading. Slides are then mounted with a glass coverslip and sealed with clear nail polish.

#### Image acquisition

All slides of fluorescently labeled, fixed single fibers are imaged using a Leica DMi8 Thunder wide field fluorescence microscope equipped with Leica’s LED8 illuminator and K8 camera (6.5 μm pixels). Fibers are first visually characterized as either Type I (MyHC-1+) or Type II (MyHC-1-) using the HC PL FLUOTAR 10x/.32 objective in the TRITC channel (excitation and emission filters: 554/24, 594/32) at 25% illumination intensity and an exposure time of 200 ms.

Once identified based on fiber type, image stacks of myonuclei on entire single fibers spanning multiple fields are acquired at higher magnification and resolution using a HC PL FLUOTAR 20x/0.4 objective in the DAPI channel (excitation and emission filters: 407/375, 450/20) at 27% illumination intensity, exposure time of 100 ms, and 2.7 μm step size (SVI Nyquist calc., https://svi.nl/Nyquist-Calculator). Image stacks are then stitched in the LAS X software for downstream analysis.

#### 3D myonuclear quantification using IMARIS

Raw, merged z-stacks of single fibers are first converted to the .ims format before being imported into IMARIS for further analysis. Prior to myonuclear quantification, images are processed with a background subtraction algorithm (filter width = 10 μm) to enhance myonuclear signal relative to the fiber background. Myonuclei from multiple single fibers are measured using a Line Profile within IMARIS, from which the average diameter of a myonucleus is estimated to be 10 μm, resulting in a background subtraction filter width of 10 μm.

IMARIS’ Spots module is used for accurate myonuclear quantification. First, the Estimated XY Diameter of the feature object is input as 10 μm based on previously described myonuclear measurements. IMARIS’ Spots prediction is then thresholded using IMARIS’ Quality parameter to obtain myonuclear counts. Total counts are manually quality-controlled by including counts of myonuclei not picked up by the Spots algorithm due to weak fluorescence signal, as well as manually excluding Spots that are incorrectly placed on aberrant DAPI signal or on nuclei outside of the muscle fiber.

#### 3D fiber length measurement

Fiber length is obtained using IMARIS’ Filaments module. First, a mask of the entire fiber is created using the Surface Creation module. The whole single fiber is segmented on the DAPI channel using a smoothing width of 15 μm, then masked and binarized by setting the pixel value within the segmented surface to 1.00. These masking steps in the Surface Creation module result in a duplicated DAPI channel with consistent signal throughout the entire single muscle fiber in all 3 dimensions.

IMARIS uses representative Seed Points placed along the fiber as a guide to estimate the path that the Filament should follow along the DAPI signal. This method optimizes both the source channel with a smoothed, binarized DAPI signal, as well as optimal Seed Points that are manually placed along the fiber. After indicating the Thinnest Diameter of the object of interest as 100 μm (approximate diameter of a muscle fiber), IMARIS’ default Seed Points are removed. Then, in Slicer View with a Slice Width of 10 μm, new Seed Points are manually placed at the center of the fiber from end-to-end. This results in a smoothed Filament that follows the center of the muscle fiber. Fiber length is measured by obtaining the Filament length in microns.

#### 2D analysis of myonuclear shape, position, orientation, and co-localization

3D segmentation using 20x widefield z-stacked images in IMARIS allows us to efficiently quantify myonuclear density from a large number of fibers; however, when attempting to 3-dimensionally render myonuclear morphology from these images, Z-axis elongation and excessive light scattering distort the true shapes of the myonuclei. This distortion, an inherent issue with widefield systems, results in nuclei appearing artificially elongated in the z-dimension.

#### Overview of the FIJI macro pipeline

The macro is designed to handle batch processing of Leica .lif files and automatically parses sample metadata (SubjectID, timepoint, leg) to organize outputs. It runs fully automated in FIJI macro language batch mode and includes the following steps:

1. Image import and preprocessing

Each .lif file is opened using Bio-Formats. The macro selects only those series labeled “Merged” for analysis. Prior to image processing, a raw TIFF of the z-stack is saved downstream z-plane identification of objects. A rolling ball background subtraction (radius = 30 pixels, 9.87 μm, the average diameter of a myonucleus) is applied to the z-stack to enhance nuclear contrast.

2. Standard deviation Z-projection

The macro generates a 2D projection using the “Standard Deviation” method, which accentuates objects that differ in mean intensity across z-slices, which is ideal for identifying myonuclei. A median filter (radius = 1 pixel) is applied to reduce noise before automatic thresholding via Otsu’s method. The image is binarized and converted to an 8-bit mask to ensure compatibility with downstream processing. These projections are saved under a “STDIP” directory for each sample.

3. Skeletonization workflow

The projected image is duplicated, blurred and thresholded again with Otsu. This step transforms the DAPI mask created in Step 2 into a representative mask of the overall fiber shape. FIJI’s Analyze Particles is then used to isolate objects larger than 70,000 pixels^2^ (23,020 μm^2^), thus segmenting out any smaller objects remaining in the image. This mask is converted to 8-bit binary to guarantee compatibility with the Skeletonize function in FIJI. The resulting 1-pixel-wide skeleton is saved under a “Skel” directory.

4. File structure and organization

Outputs are organized automatically by subject, timepoint, and leg in nested directories.

#### Python-based myonuclear analysis

##### Input structure and parsing

Each fiber is represented by three co-registered images stored within standardized subdirectories:

• *STDIP/* - binary standard-deviation projection image marking nuclear masks;
• *Skel/* - 2D representative fiber skeleton image;
• *TIFs/* - z-stacked 3D image with dimensions (Z, Y, X).

The parser is robust to naming variants and missing files.

##### Parameterization

All adjustable constants—such as pixel-to-micron conversion, z-axis scaling, area filtering limits, and clustering radius—are stored in a *Params* dataclass. This allows all analysis steps to reference a single structured parameter object and supports command-line overrides; encapsulating parameters in a dataclass makes the pipeline reproducible and portable across imaging conditions and magnifications.

##### Image loading and preprocessing

Utility functions handle file input and preparation:

• *_imread()* loads TIF/TIFF files using scikit-image.
• *_to_bool()* ensures binary STDIP masks are converted to Boolean arrays.
• *_label_mask()* labels nuclei in the binary image, optionally removing objects smaller than a given minimum area (min_area_px = 200, 65.831 μm^2^).

##### Nuclear morphology measurements

The function *measure_nuclei_from_binary()* computes morphometric descriptors for each labeled nucleus of the STDIP image: area, perimeter, centroid coordinates, major/minor axis lengths, and orientation. Derived quantities include:

• *Aspect ratio* = minor/major axis length. A value of 1 indicates a perfect circle; values approaching 0 indicate an ellipse.
•*Shape class:* To categorize nuclei by geometric eccentricity, we established discrete boundaries: Elongated (< 0.55), Intermediate (0.55—0.80), and Circular (> 0.80). These thresholds are derived from the empirical frequency distribution of the aspect ratios within our dataset, identifying natural clustering to distinguish compressed or obliquely sectioned nuclei from those with high circularity. This threshold should be empirically determined based on research context/model.
• *Orientation* (deg) on 0–180° scale.

All measurements are then converted from pixel to micrometer units (pixel_size_xy_μm = 0.3291532 for 20x objective), specific to imaging protocol. Annotated mask overlays are also generated for quality control.

##### Z-position assignment and z-consistency filtering

Because widefield imaging introduced substantial Z-axis elongation artifacts, Z dimensions from microscope metadata could not be used for reliable 3-dimensional measurements or spatial clustering. To mitigate this distortion, the function *compute_mean_z_for_labels()* estimates the effective Z-position directly from the intensity data. For each pixel, the index of maximal fluorescence intensity (z-argmax) is identified to produce a per-pixel map of maximal signal depth. For each nucleus, the mean Z-position is accepted only when the within-object standard deviation (Z-STD) of these indices satisfies a dynamic consistency filter.

To distinguish valid biological structures from vertically overlapping artifacts or optical “flare,” the pipeline applies the following logic:

• *Planar Nuclei*: Objects with larger XY footprints are restricted to a strict Z-STD ≤ 2.0 to ensure they represent a single focal plane.
• *Vertical Nuclei:* To prevent the disproportionate exclusion of nuclei oriented perpendicular to the imaging plane (e.g., at the lateral fiber boundaries), a more lenient threshold is applied to objects with small XY areas (< 500 pixels, 164.5 μm^2^). This accounts for the natural axial span of a “vertical” nucleus while still rejecting non-biological projection artifacts.

This dual-threshold approach yields a robust *Mean_Z_um* measure for all included nuclei and improves downstream 3D spatial analysis. Both included and excluded nuclei are retained in separate CSV files, ensuring full traceability of the filtering process.

##### Relative myonuclear orientation

While the preceding morphometric measurements (Section 4) describe nuclear orientation relative to the global x-y image axes, this does not capture the true alignment of nuclei with respect to individual muscle fibers, whose trajectories may curve or vary across the tissue section. To resolve this, the function *skeleton_angles_near_nuclei()* estimates the dominant local direction of each myofiber in the vicinity of every nucleus.

For each nuclear centroid, the algorithm identifies all skeleton pixels within a fixed radius and applies Principal Component Analysis (PCA) to their (x, y) coordinates. The first principal component corresponds to the dominant local axis of the fiber segment. Nuclear orientation, originally measured relative to the image x-axis, is then compared to this local fiber axis to compute a relative alignment angle.

This relative angle is expressed on a 0°–90° scale, where 0° denotes perfect alignment of the nucleus with the fiber axis and 90° indicates perpendicular orientation. This approach provides a curvature-aware measure of nuclear alignment that reflects local myofiber structure rather than global image geometry.

##### Distance to fiber skeleton

To quantify nuclear positioning relative to the fiber core, *compute_distance_to_skeleton()* calculates the Euclidean distance from each nucleus centroid to the nearest skeleton pixel. This produces a *DistanceToSkel* variable (μm) for every nucleus. This metric enables downstream classification of “central” nuclei and supports nuclear chain analysis.

##### 3-dimensional clustering of nuclei

To identify spatial clusters of myonuclei within individual muscle fibers, we apply the Density-Based Spatial Clustering of Applications with Noise (DBSCAN) algorithm via the *sci-kit learn* library. DBSCAN was chosen for its ability to identify clusters of arbitrary shape based on local point density while effectively segregating outliers. Clustering is performed using 3-dimensional myonuclear centroid coordinates (Position X, Position Y, Position Z).

The algorithm is parameterized with a *min_samples* value of 2 (defining a cluster as at least two nuclei) and an *eps* value of 20 μm. As 10 μm is the average diameter of a single nucleus, an *eps* value of 20 μm corresponds to the diameters of two nuclei. Therefore, a cluster is defined as two or more nuclei that are not more than 20 μm (two nuclei) apart from one another. This parameter can be adjusted based on how the user would like to define clustering in their model. For example, Increasing the eps by 5 μm will widen the search range, resulting in fewer clusters with more nuclei in each. Similarly, decreasing the eps by 5 μm will decrease the search range, resulting in more clusters with fewer nuclei in each.

Cluster labels assigned by DBSCAN are then added to the dataset with unclustered nuclei (noise) labeled as -1.

##### Nearest-neighbor analysis

To further characterize the local nuclear microenvironment, the pipeline computes 3- and 5-nearest-neighbor (NN3/NN5) features for each included nucleus using 3D Euclidean distances. Extracted contextual features include:

• *NNk_MeanDist_um* – local nuclear packing density
• *NNk_MeanAspectRatio* – neighborhood shape profile
• *NNk_Frac_Circular / Elongated / Intermediate* – shape class composition
• *NNk_Frac_Parallel* / Perpendicular / IntermediateOrientation – orientation coherence within the microenvironment
• *NNk_MeanRelAngle_deg* – neighbor alignment relative to local fiber axis

These metrics quantify nuclear neighborhood structure and support analysis of “myonuclear footprint” organization.

##### Fiber diameter profiling

The function *compute_fiber_width_profile()* samples diameters along the skeleton at fixed intervals (default 100 μm). Diameters are estimated by projecting included nuclei positions perpendicular to the local axis and measuring left–right separation. This yields a diameter profile and summary statistics (mean, min, max, SD) to inform downstream volume analyses.

##### Myonuclear domain volume

The pipeline also computes a volume-based myonuclear domain for each fiber:

• *FiberVolume* (μm^3^) = π · (MeanDiameter/2)^2^ · IMARISFiberLength
• *DomainVolume_um3_Python* = FiberVolume / PythonMyonuclei
• *DomainVolume_um3_IMARIS* = FiberVolume / IMARISMyonuclei (if available)

This provides a volumetric estimate of the cytoplasmic territory associated with each myonucleus.

##### Biopsy-level summaries

For each fiber, derived results are merged with *IMARIS* master file metrics (fiber length, nuclei counts), if available. Additional biopsy-level variables are computed:

• Nuclear counts (PythonMyonuclei, IMARISMyonuclei)
• Diameter statistics and FiberVolume
• Myonuclear domain volume (Python/*IMARIS*)
• Neighbor microenvironment metrics (fiber-level means of NN3 features)
• Shape and orientation distributions
• Cluster# (3D DBSCAN clusters)
• Nuclear chain and central-nuclei counts

##### Batch orchestration and outputs

Helper functions automate batch processing across subjects, timepoints, and legs. Each subject receives a workbook *(<Subject>_results.xlsx)* with one sheet per timepoint, preserving column order and preventing Excel auto formatting of fiber IDs. Per fiber outputs include:

• *nuclei_results.csv* (included nuclei)
• *excluded_nuclei.csv* (excluded objects)
• *fiber_width_profile.csv* (diameter sampling)
• *overlay.png* (annotated mask image)

Biopsy level summaries integrate Python metrics with outputs, providing a unified dataset for downstream statistical analysis.

### Quantification and statistical analysis

Analyses are performed in GraphPad Prism 10.4.2 and Python (custom pipeline). Method comparisons and validations use Bland–Altman analysis to assess agreement and Pearson correlation to quantify association between methods. Average percent error is reported for accuracy comparisons. Intraclass correlation coefficients (ICC) are computed for intra- and inter-rater reliability.
